# A scalable 12-week exercise and education programme reduces symptoms and improves function and wellbeing in people with hip and knee osteoarthritis

**DOI:** 10.3389/fresc.2023.1147938

**Published:** 2023-04-26

**Authors:** Jemma L. Smith, Aidan Q. Innes, Danielle S. Burns, Davina Deniszczyc, James Selfe, Stephen MacConville, Kevin Deighton, Benjamin M. Kelly

**Affiliations:** ^1^ Research, Outcomes and Data Science, Nuffield Health, Epsom, United Kingdom; ^2^ Department of Health Professions, Faculty of Health and Education, Manchester Metropolitan University, Manchester, United Kingdom

**Keywords:** osteoarthritis, exercise, wellbeing, joint, knee, hip

## Abstract

**Introduction:**

Osteoarthritis is a chronic musculoskeletal condition that impacts more than 300 million people worldwide, with 43 million people experiencing moderate to severe disability due to the disease. This service evaluation provides the results from a tailored blended model of care on joint health, physical function, and personal wellbeing.

**Methods:**

1,593 adult participants with osteoarthritis completed the Nuffield Health Joint Pain Programme between February 2019 and May 2022. The 12-week programme included two 40-min exercise sessions per week. All exercise sessions were conducted face-to-face and were followed by 20 min of education to provide information and advice on managing osteoarthritis.

**Results:**

The 12-week joint pain programme significantly improved Western Ontario and McMaster Universities Osteoarthritis Index (WOMAC) global scores (Week 0: 37.5 [17.2]; Week 12: 24.0 [16.6]; *p* < 0.001), as well as subscales for pain (Week 0: 7.6 [3.7]; Week 12: 4.9 [3.7]; *p* < 0.001), function (Week 0: 26.0 [13.0]; Week 12: 16.3 [12.4]; *p* < 0.001), and stiffness (Week 0: 3.9 [1.6]; Week 12: 2.8 [1.7]; *p* < 0.001). Significant improvements in health-related outcomes including systolic and diastolic blood pressure (Week 0: 139 [18] mmHg; Week 12: 134 [17] mmHg, and Week 0: 82 [11] mmHg; Week 12: 79 [19] mmHg; both *p* < 0.001), body mass index (Week 0: 29.0 [4.5] kg/m^2^; Week 12: 28.6 [4.4] kg/m^2^; *p* < 0.001), waist to hip ratio (Week 0: 0.92 [0.23]; Week 12: 0.90 [0.11], *p* < 0.01) and timed up and go (Week 0: 10.8 s [2.9]; Week 12: 8.1 s [2.0]; *p* < 0.001) were also observed. On completion of the joint pain programme, participants also reported significant improvements in all assessed aspects of self-reported wellbeing (all *p* < 0.001).

**Discussion:**

With reductions in physical symptoms of osteoarthritis and improvements in personal wellbeing, the joint pain programme delivered by personal trainers in a gym-setting offers a nationally scalable, non-pharmacological treatment pathway for osteoarthritis.

## Introduction

Osteoarthritis (OA) is a chronic musculoskeletal condition characterised by stiffness and pain in joints. Globally, OA impacts 3.3% to 3.6% of the population and with 43 million people experiencing moderate to severe disability as a result of OA, it is the 11th most debilitating disease worldwide (1). Though any joint is susceptible to OA, the hip and the knee are the most commonly affected joints, with over 300 million cases reported worldwide in 2017 (2).

There are numerous risk factors for developing OA including older age, female gender, obesity, anatomical factors, muscle weakness, and joint injury (1). Such risk factors, whether in isolation or combined, induce a series of cellular changes and biomechanical stresses that contribute to the degeneration of the joint. Some of the mechanisms underlying OA include synovial inflammation and hypertrophy, articular cartilage damage and subchondral bone thickening. While the presentation and progression of OA varies from person to person, the most common physical symptoms are joint pain, stiffness, and mobility problems. Such symptoms are also related to impaired quality of life and poor psychological outcomes (3).

There are several recognised approaches to managing OA which often include the combination of self-management and pharmacological treatments. Pharmacological interventions such as non-steroidal inflammatory drugs (NSAIDs), opioids and analgesics can be effective at relieving pain and improving function in people with hip and knee OA (4). Although effective, NSAIDs have long been associated with an increased risk of gastrointestinal, cardiovascular and renal harms when compared with placebo (5). Side effects are elevated in individuals with co-morbidities, of which 67% of people with OA have at least one other chronic condition (6), positioning NSAIDs as inappropriate for the majority of people with OA. Furthermore, pharmacological solutions do not slow down the degradation of joints and worsening of the disease. As such, other management options for OA exist ranging from minimally invasive treatments to intensive surgical interventions. Whilst surgical intervention, such as total joint replacement, is largely successful in improving outcomes for people with OA (7), this is often a costly last resort for individuals suffering with severe life-limited OA. Surgical intervention also has much greater risks than other less invasive interventions. Evidence demonstrates that less invasive OA interventions such as intra-articular injections are effective at improving physical function, provide pain relief, and bolster quality of life (8, 9).

Exercise is advocated by international guidelines for the management of OA including National Institute for Health and Care Excellence (NICE) and the Osteoarthritis Research Society International (OARSI) (10). In clinical practice, exercise is often prescribed, either in isolation or combined with physical agent modalities such as intra-articular injections and NSAIDs (11). Evidence demonstrates that exercise interferes with the progression of OA by affecting pathological changes such as articular cartilage degradation, apoptosis, and the inflammatory response. For OA, many types of exercise training such as aerobic exercise, strength training, neuromuscular training and balance training can relieve pain, as well as improve muscle strength, physical functioning, and quality of life (12). Although many exercise training modalities have been shown to benefit OA, exercise prescription should be tailored to the individual person with regards to the frequency, intensity, type and duration in order to ensure compliance, the mastery of new skills and injury prevention (12). Exercise training offers a tangible, low-cost alternative to pharmacological approaches in the management of OA, regardless of disease severity.

In OA, education alongside exercise therapy is thought to improve pain and function when compared with exercise therapy alone. Several approaches to education have been utilised, ranging from basic knowledge acquisition to condition-specific self-management skill development (13) and delivery methods including lectures, group-based sessions, self-directed materials or telephone calls and home visits. While there is a lack of certainty regarding the optimal content and/or delivery methods for people with OA, across a number of musculoskeletal conditions education is shown to improve illness perception (14), self-efficacy (15), and fear-avoidance behaviours (16). Further, for severe OA, evidence illustrates that exercise and education before total hip and knee replacement can be effective at improving pre-surgical health and early recovery (17).

Several evidence-based services for people with hip and knee OA are available worldwide. The Good Life with osteoArthritis in Denmark (GLA:D) programme is a structured treatment program for OA, consisting of exercise therapy and evidence-based education, which has been shown to elicit improvements in pain and objective function in Denmark, Canada, and Australia (18). In the UK, the ESCAPE-pain programme is delivered by physiotherapy staff in the National Health Service (NHS) for groups of eight to ten people (19). It is important to note that waiting times for elective NHS orthopaedic procedures have risen considerably due to the COVID-19 pandemic (20). As such, an evidence-based program, delivered in a community gym setting rather than the NHS, has the potential to reduce some of the strain on the healthcare system in the UK, and widen access to OA services.

The current service evaluation aims to examine the effect of a community gym-based exercise and education program on symptoms of pain, function, and stiffness and general health and personal wellbeing in participants with OA of the hip and/or knee.

## Materials and methods

### Study design and participants

This service evaluation was conducted according to the guidelines laid down in the Declaration of Helsinki, and all procedures were approved by the Ethics Advisory Committee at Manchester Metropolitan University (Ref: 11654). Participants were able to enrol into the JPP at session 1 online *via* self-referral or referral from an NHS practitioner. All participants reported in the results provided written informed consent for the inclusion of outcome data in external publications.

This service evaluation used baseline and follow-up data (at 12 weeks) from 1,593 people with hip and/or knee OA undertaking the Nuffield Health Joint Pain Programme (JPP) between February 2019 and May 2022. A full overview of the inclusion and exclusion criteria for the JPP is provided in Table 1.

**Table 1:** Study inclusion and exclusion criteria.

Inclusion Criteria	Exclusion Criteria
Previous diagnosis of OA at the hip, knee or both joints for at least one year	Participants already undergoing treatment and/or physiotherapy for their OA *via* the NHS or private healthcare
18 years of age and over	Have unmanaged medical conditions that contraindicate unsupervised exercise
Must have access to the internet and smartphone/tablet/personal computer (with adequate technology literacy)	Have had joint-related surgery in the previous three months
Access to transport for session attendance	

### Joint pain programme

The JPP aims to improve the joint health, physical function, and personal wellbeing of people with OA of the hip and/or knee. The JPP is a 12-week group-based, face-to-face programme consisting of 2 × 1-h sessions per week (24 in total), with a maximum of 10 participants permitted per group, Table 2 details the intervention according to the Template for Intervention Description and Replication checklist.

**Table 2:** Overview of joint pain programme according to the TIDieR checklist.

Item No	Item
**Brief name**	
1	Joint Pain Programme
**Why**	
2	The JPP aims to improve joint health, physical function, and personal wellbeing of people with OA of the hip and/or knee.
**What**	
3	Participants had access to a web-based joint-pain-hub (available here https://www.nuffieldhealth.com/joints-content-hub) which contained educational information, advice, and tips for managing joint pain. Participants were also provided with a physical copy of the JPP journal, which included the educational activities of the JPP and a log to document personal goals and progress.
4	Each week, participants engaged in 1 × 40-min circuit-based exercise session and 1 × 40-min exercise class. The remaining 20-min of each session was educational including information and advice on managing OA. Educational components included an overview of OA, the importance of exercise in OA, perception of pain as well as weight management, emotional wellbeing, and recovery strategies.
**Who provided**	
5	All sessions were delivered by Nuffield Health personal trainers who had received specialist training in joint pain conditions, exercise modalities for joint pain, and methods of effective data collection. On successful completion of the JPP course, the personal trainer has access to an online platform containing relevant materials for delivering the programme. This ensures that the delivery of the JPP remains consistent in Nuffield Health Fitness and Wellbeing Centres across the UK.
**How**	
6	The JPP is a group-based, face-to-face programme with a maximum of 10 participants permitted per group.
**Where**	
7	The JPP was conducted at 31 Nuffield Health Fitness and Wellbeing Centres, all of which were registered with the Care Quality Commission (England) or the Care Inspectorate (Scotland). Nuffield Health Fitness and Wellbeing centres are commercial gyms, available to the public.
**When and How Much**	
8	The joint pain programme consisted of 2 × 1-h sessions per week (24 in total). Participants completed one circuit-based activity session and one complimentary exercise class per week. Participants were encouraged to exercise to a light-to-moderate intensity, ensuring not to exceed the restraints of their joint pain condition, and a rest ratio of 1:1 or 1:2 was employed. An overview of exercise selection, repetitions, sets and duration is presented in Supplementary Table S2.
**Tailoring**	
9	A person tailored approach was utilised to determine exercise selection, duration, intensity, and work to rest ratio, considering the individuals joint pain condition, exercise capacity and capabilities.
**Modifications**	
10*	The joint pain programme was not modified throughout the 12 weeks.
**How well**	
11	Adherence to the joint pain programme was logged by the personal trainers leading the session manually.
12*	The median and interquartile range for session attendance was 21 (20–24) out of the 24 sessions, with 25.6% of participants attending all sessions.

Each week, participants engaged in 1 × 40-min circuit-based exercise session and 1 × 40-min exercise class. Sessions were led by a personal trainer and focused on progressively improving: (1) cardiovascular fitness, (2) joint and functional mobility, (3) joint stability and balance, and (4) strength. Participants engaged in a cardiovascular based session in week 1, a mobility session in week 2, balance in week 3, and strength in week 4, the focus of these sessions were then repeated in weeks 5–8 and weeks 9–12. Activity-based exercise sessions mirrored the target exercise modality of the circuit-based session. An overview of these sessions is provided in Supplementary Table S2.

A person-tailored approach was utilised to determine exercise selection, duration, intensity, and work to rest ratio, considering the joint pain condition, exercise capacity and capabilities of each participant. Participants were encouraged to exercise to a light-to-moderate intensity, ensuring not to exceed the restraints of their joint pain condition, completing 10–15 repetitions of exercises for 2–4 sets with a rest ratio of 1:1 or 1:2.

The remaining 20-min of each session was dedicated to participant education including information and advice on managing OA. Educational components included an overview of OA, the importance of exercise in OA, perception of pain as well as weight management, emotional wellbeing, and recovery strategies. A weekly breakdown of the JPP educational activities is provided in the Supplementary Materials.

Participants had access to a web-based joint-pain-hub (available here https://www.nuffieldhealth.com/joints-content-hub) which contained educational information, advice and tips for managing joint pain. Participants were also provided with a physical copy of the JPP journal, which included the educational activities of the JPP and a log to document personal goals and progress.

The JPP was conducted at 31 Nuffield Health Fitness and Wellbeing Centres, all of which were registered with the Care Quality Commission (England) or the Care Inspectorate (Scotland). Nuffield Health Fitness and Wellbeing centres are commercial gyms, available to the public. For the JPP, participants were provided with a free 12-week membership to their local centre, and a charge of £2 per session of the JPP was required. Upon completion of the JPP, participants were given the option of a further 3-months access for £16 per month if they wanted to continue using the Fitness and Wellbeing Centre. Alongside the 12-week programme, participants were encouraged to continue being physically active and to exercise, using their free gym membership.

### Personal trainer development training

All sessions were delivered by Nuffield Health personal trainers who had received specialist training in joint pain conditions, exercise modalities for joint pain, and methods of effective data collection. On successful completion of the JPP course, the personal trainer was given access to an online platform containing relevant materials for delivering the programme. This ensured that the delivery of the JPP remained consistent in Nuffield Health Fitness and Wellbeing Centres across the UK.

### Outcome measures

Outcome data were collected for all participants at baseline (Week 0) and on completion of the JPP at Week 12. Data was objectively measured by the personal trainer or self-reported by the participant and stored on a web-based SharePoint platform.

### Joint pain

Joint pain was assessed using the Knee Osteoarthritis Outcomes Survey (21) and/or the Hip Osteoarthritis Outcomes Survey (HOOS, 22). The KOOS and HOOS are instruments that assess a patient's opinion about their affected joint and associated problems, and consist of questions regarding Pain, Symptoms, Activities of Daily Living Function, Sport and Recreation, and Quality of Life. The KOOS and HOOS produce a percentage score from 0 to 100, with 0 representing extreme problems and 100 representing no problems at all.

Both the KOOS and HOOS are extensions of the Western Ontario and McMaster Universities Osteoarthritis Index (WOMAC), a widely used health status measure assessing pain, stiffness, and function for people with OA of the hip and/or knee. Global WOMAC scores range from 0 to 96, pain scores from 0 to 20, stiffness from 0 to 8, and function from 0 to 68. WOMAC scores are the inverse of KOOS and HOOS scores, with 0 representing no problems at all and the higher scores representing extreme problems. Reductions in WOMAC scores demonstrate an improvement in OA.

WOMAC global scores, and subscale scores for pain, stiffness, and function were calculated by transforming the data from relevant KOOS and HOOS items. The KOOS and HOOS questionnaires, scoring manual, and user's guides can be downloaded from http://www.koos.nu.

### Functional mobility

Functional mobility and balance were assessed using the timed up and go test, which is a test recommended by the OSARI as a performance-based test for hip and knee OA (23). The timed up and go test is a simple screening test that assesses the time taken for a person to go from a seated position, to standing, walking 3 metres, walking back to the chair, and sitting down. Persons who take ≥12 s to complete the timed up-and-go are at an elevated risk of falling.

### General health

General health related outcomes including body mass index (BMI), waist-to-hip ratio, blood pressure, resting heart rate and finger prick glucose tests were also collected.

### Physical activity

Physical activity was assessed using a question about how many hours per week that the participant was physically active, within the last three months. Responses included “less than 1 h a week”, “1–2 h a week”, “2–3 h a week”, or “more than 3 h a week”.

### Personal wellbeing

Personal wellbeing was assessed using the ONS4 questionnaire (24). This comprises four questions, with each item answered on a scale of 0 to 10, where 0 is “not at all” and 10 is “completely”. Measures cover life satisfaction, life being worthwhile, happiness, and anxiety. Higher scores of life satisfaction, life being worthwhile, and happiness indicate better personal wellbeing. Higher scores of anxiety indicates poorer personal wellbeing.

### Statistical analyses

The primary analyses were conducted in 1,593 participants who completed the JPP and provided outcome measures at baseline (Week 0) and post-intervention (Week 12). Results are provided separately for participants with hip and knee OA, as well as for all participants combined for measures that were not specific to the hip or knee joint.

Graphical representations of the results are provided as mean [95% confidence interval (CI)]. Descriptions of data in the text for individual timepoints are provided as mean (SD), with the difference between timepoints provided as mean (95% CI). Paired samples *t*-tests were used to determine significant differences between timepoints for general health, WOMAC, and personal wellbeing outcomes. The McNemar chi-squared test was used to determine significant differences between timepoints for self-report physical activity levels. Two-sided 95% CIs were used for all analyses and all significance tests were performed at the 5% alpha level.

The primary outcome for the service evaluation was the WOMAC function subscale score, with the minimum clinically important difference (MCID) determined from the literature as a 26% decrease for participants with knee OA and a 21.1% decrease for participants with hip OA (25). The sample size for the present evaluation was comparable to the work by Tubach and colleagues (25) which determined the MCID and was therefore deemed to be adequately powered to detect this change. The 95% CIs of the change in WOMAC function scores between timepoints were compared against the MCID. Where the 95% CIs of the difference between timepoints exceeded the MCID, this demonstrated that the change was significantly greater than the MCID.

All analyses were performed using R version 4.1.2 (26).

## Results

A total of 2,571 participants were assessed for eligibility to participate in the JPP. Of these, *n* = 615 did not meet the inclusion criteria (*n* = 375 did not have access to transport for session attendance and *n* = 240 were already receiving OA treatment or rehabilitation). An additional *n* = 317 did not commence the JPP due to attrition between initial registration and being invited onto the programme, largely due to gym closures and a consequent pause in the programme during the COVID-19 national lockdown. An additional *n* = 46 participants did not consent to publication of outcome measures. Complete case analysis was conducted for the 1,593 participants who completed the programme and the relevant outcome measures at Weeks 0 and 12.

### Participant Characteristics

All participants had previously been diagnosed with OA of the hip, knee or both joints for at least one year, with a mean self-reported pain duration of 10.2 years (9.9 SD). The most frequently reported site of pain was the knees (66%), followed by the hips (32%) and both joints (2%). On programme enrolment, participants had a mean age of 64 (9 SD) years. The majority of participants were female (84%) and were retired (61%). Further demographic information is provided in the Supplementary Materials.

### Adherence

The median and interquartile range for session attendance was 21 (20–24) out of the 24 sessions, with 25.6% of participants attending all sessions. There were no adverse events such as minor injuries reported across the duration of the programme.

### Outcomes

**Figure 1:**
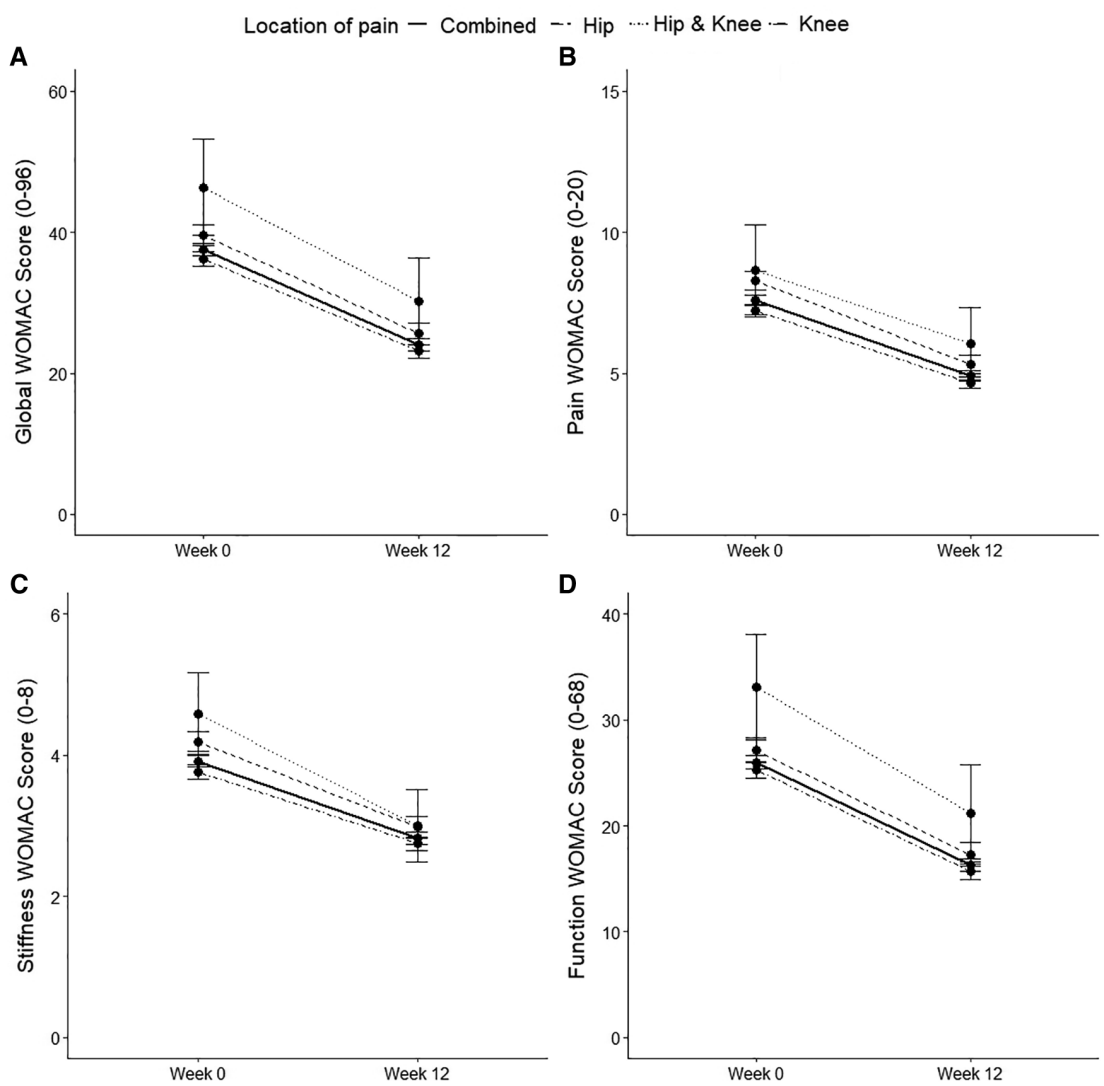
Baseline and Week 12 values for global WOMAC scores (**A**); pain score (**B**); stiffness score (**C**); and function score (**D**). Values are presented as mean (95% CI). Higher scores indicate worse symptoms for WOMAC scores. Differences between time points were analysed using paired *t*-tests. For all groups, all *p* < 0.001. All patients Combined: *n* = 1593; Hip only: *n* = 505; Knee only: *n* = 1,055; Hip & Knee: *n* = 33.

The 12-week JPP significantly improved function scores for knee OA, hip OA and when these subgroups were combined (Figure 1D; all *p* < 0.001). Additionally, the 95% CIs for these improvements exceeded the MCID for people with knee OA (CI: 40.4% to 35.4%) and hip OA (CI: 39.8% to 32.6%). Significant improvements in global WOMAC scores, as well as subscale scores for pain and stiffness were also observed (Figure 1; all *p* < 0.001). Changes in individual components of the KOOS and HOOS questionnaire scores from baseline to Week 12 are available in the Supplementary Materials.

**Figure 2:**
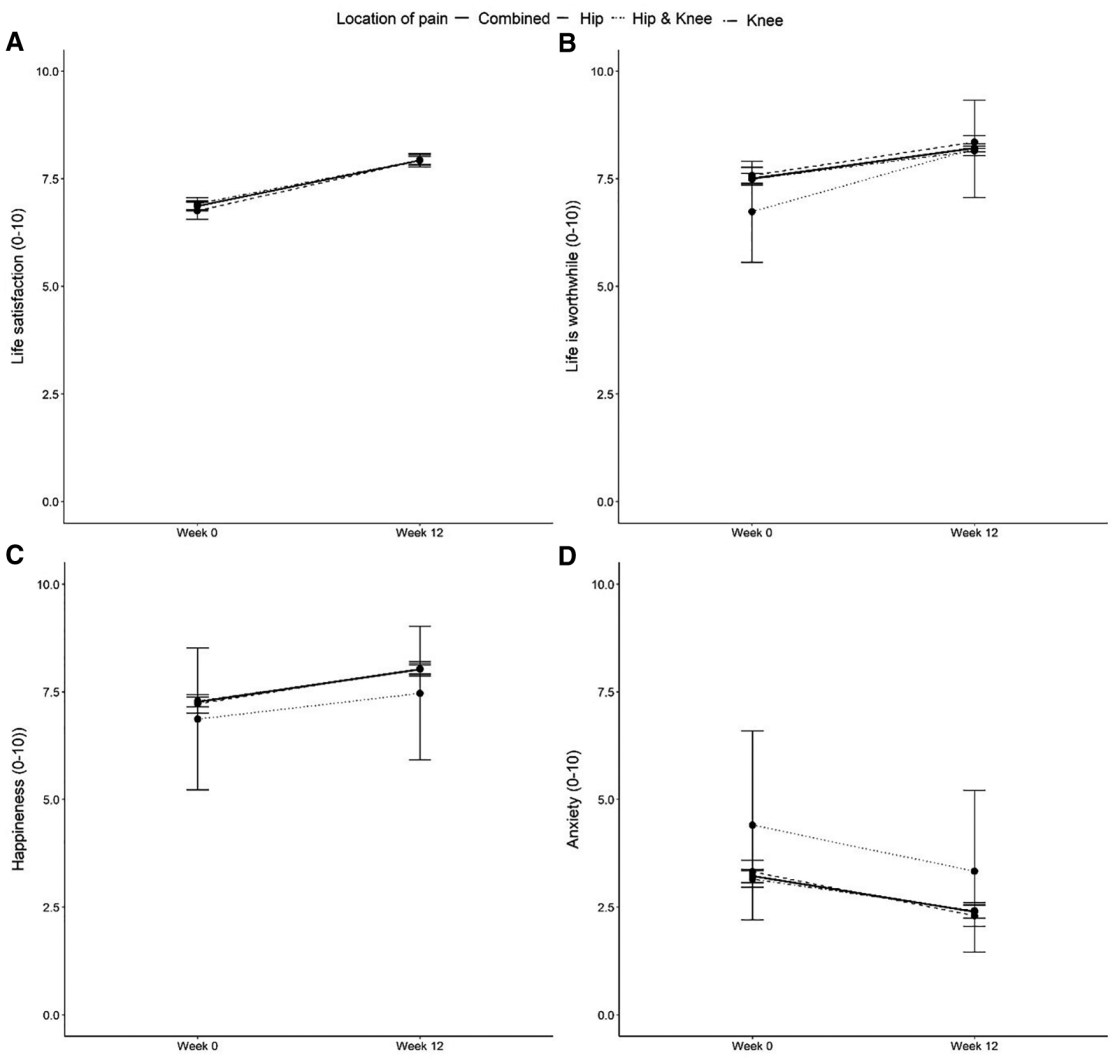
Baseline and Week 12 values for ONS-4 self-reported life satisfaction (**A**), life being worthwhile (**B**), happiness (**C**) and anxiety scores (**D**). Values are presented as mean (95% CI). Lower scores indicate worse health for ONS4 life satisfaction, life is worthwhile and happiness scores. Higher scores indicate worse health for ONS4 anxiety. Differences between time points were analysed using paired t-tests. For all groups, all *p* < 0.001. Combined: *n* = 1278; Hip scores: *n* = 413; Knee scores: *n* = 850; Hip & Knee: *n* = 15.

Significant improvements in health-related outcomes including systolic and diastolic blood pressure, body mass index, timed up and go, and waist to hip ratio were also observed (Table 3). No significant differences were observed for fasting glucose and resting heart rate. Participants reported significant increases in physical activity levels at programme completion (*p* < 0.001). These improvements were consistent for participants with hip OA, knee OA and those with OA at both joints, with the changes for these groups presented separately in the Supplementary Materials. All assessed aspects of self-reported wellbeing were measured using the ONS4. Upon programme completion, participants reported significant improvements in all assessed aspects of self-reported wellbeing from Week 0 to Week 12 (all *p* < 0.001; Figure 2).

**Table 3:** General health pre- and post-JPP.

	Week 0	Week 12	Δ Week 0 to 12
Systolic Blood Pressure (mmHg)	139 [18]	134 [17]	5 [0.74]***
Diastolic Blood Pressure (mmHg)	82 [11]	79 [19]	3 [0.44]***
Body Mass Index (kg/m^2^)	29.0 [4.5]	28.6 [4.4]	0.4 [0.08]***
Fasting Glucose (mmol/L)	4.8 [2.3]	4.8 [2.7]	0 [0.1]
Resting Heart Rate (beats per minute)	71 [12]	72 [11]	1 [0.48]
Timed Up and Go (seconds)	10.8 [2.9]	8.1 [2.0]	2.7 [0.13]***
Waist To Hip Ratio	0.92 [0.23]	0.90 [0.11]	0.02 [0.01]**

Values for each timepoint are presented as mean (SD), while delta values are presented as mean (95% CI). Higher scores indicate a worse health state. Differences between time points were analysed using paired *t*-tests. ***p < 0.001, **p < 0.01, *p < 0.05. Systolic Blood Pressure: *n* = 1,492; Diastolic Blood Pressure: *n* = 1,492; Body Mass Index: *n* = 977; Fasting Glucose: *n* = 978; Resting Heart Rate: *n* = 1,487; Timed Up and Go: *n* = 1,478; Waist to Hip ratio: *n* = 1,428; *N* values for Δ Week 0 to 12.

## Discussion

This service evaluation demonstrates that the Nuffield Health JPP significantly reduces OA symptoms for participants with OA of the knee and/or hip. The outcomes demonstrate significant improvements in joint pain and stiffness, personal wellbeing, and clinically meaningful improvements in the primary outcome of joint function. These findings position the JPP as a viable, scalable rehabilitation model for individuals presenting with OA at the hip or knee.

The improvements in physical function in response to the JPP were demonstrated *via* a mean 9.5-point (37.9%) and a 9.8-point (36.2%) improvement in WOMAC function score for knee and hip OA, respectively, with 95% CIs exceeding the MCID to demonstrate a significantly meaningful improvement. For chronic diseases such as OA, the goal of rehabilitation is to reduce symptoms and to make the individual as independent as possible regarding daily activity. The JPP predominantly focused on increasing physical function but importantly contained elements of education regarding symptom management and healthy lifestyle modification. In a recent systematic review conducted by Sinatti and colleagues (2022), patient education was shown to be effective at reducing pain and improving function in people with OA of the hip and knee joint (27). Interestingly, the authors concluded that combining patient education with OA treatments, such as physical activity, should be encouraged given the superior improvements in outcomes when compared with physical activity alone. This is supported by the improvements in symptoms achieved following the Nuffield Health JPP, which advances these findings into a gym-based setting.

The precise mechanisms underlying the protective effects of exercise in OA are currently not well understood (28). Given the variety of exercises performed during the JPP, an array of physiological and biomechanical effects are likely responsible for eliciting improvements in pain, stiffness, and function. Weight-bearing exercise produces several joint-related benefits including improved blood and synovial fluid circulation, increased muscle size, reduced inflammation and increased joint stability (29). These adaptations may have boosted physical functioning and had synergistic effects on pain perception (30). Indeed, previous research has demonstrated tangible benefits in people's perception of health, behavioural responses to pain, and self-management strategies following engagement in exercise (31).

Consistent with the improvements in physical symptoms, significant increases in personal wellbeing were observed in response to the 12-week JPP. Participants demonstrated a mean 13% improvement in life satisfaction, a 9% improvement in life being worthwhile, and a 9% improvement in happiness scores, alongside a 26% reduction in anxiety scores. The mental wellbeing effects of exercise are well documented in OA and chronic pain, with exercise shown to augment overall mood, and reduce anxiety and depression (32, 33). Additionally, the JPP utilised a group-based exercise approach, with studies demonstrating that group exercise bolsters physical and mental health, increases social connectedness and reduces loneliness, particularly among older age adults (34, 35). As such, a synergistic effect of exercise on physiological, psychological, and sociological factors are likely responsible for the improvements in personal wellbeing.

Previous studies have shown exercise-based interventions to be more beneficial for pain relief in patients with knee OA vs. hip OA (36). In the JPP however, changes in physical and wellbeing symptoms were similar for patients with knee OA and hip OA. The disparity in findings between the present service evaluation and previous research may be attributed to several factors. First, many types of exercise have been examined in the literature, such as aerobic exercise (37), strength training (38), swimming (39), neuromuscular exercise (40), and balance training (41). Clearly there is considerable variation in exercise-based interventions for OA and with different formats of exercise unique physiological and molecular changes occur. Further, it is generally recommended that OA patients engage in exercise at least 12 times within three months when initiating a program (42). In the JPP, a total of 24 sessions were completed in that same time-period, thereby exceeding OA recommendations. Finally, evidence supports an inverse association between exercise benefits and OA severity, with greater improvements observed in milder OA compared with severe cases (36). This is relevant to the current service evaluation as participants demonstrated a mean baseline WOMAC function score of 26/68, indicative of mild to moderate disease severity. Nonetheless, the 9.7-point mean improvement in WOMAC function score at the end of the intervention is greater than that previously reported in studies with a similar participant population (43). It is important to note that OA symptom type, intensity, and frequency vary for each individual (44). Taken collectively, exercise modality and frequency, and disease severity interplay to elicit differences in OA clinical outcomes.

## Limitations

This service evaluation has certain limitations. First, a control group was not included. Therefore, we cannot directly attribute all of the observed benefits to the JPP, or understand the influence of factors such as regular social interaction with other participants or having support from personal trainers. However, in the current setting, it was deemed inappropriate to withhold the service from prospective participants for the purposes of conducting a randomised controlled trial. Previous research has demonstrated that exercise is superior to an NSAID control group for improving symptoms and quality of life in people with knee OA over a 12-week period (45), which support the benefits of the JPP observed in this service evaluation. Second, the sample were mostly older age females (83.7%). The lack of diversity in this sample illustrates the need for further work to engage with additional populations and communities. Third, improvements in OA markers were only assessed at a relatively short-time period of 12-weeks and utilising self-report joint-specific instruments. Future research into the longer-term effects of the JPP, in conjunction with further radiological, arthroscopic, and clinical biomarkers of joint health, may prove beneficial in understanding the time course response to exercise in relation to OA symptomology and disease progression.

## Conclusion

In conclusion, the Nuffield Health Joint Pain Programme produces significant improvements in physical and mental wellbeing outcomes in people with hip and knee OA. The programme itself is delivered by personal trainers rather than physiotherapists and within a gym setting. This offers a nationally scalable, non-pharmacological treatment pathway for OA. The model itself is centred around introducing people with OA to exercise and condition management, and with reductions in physical symptoms of OA, presents as a viable avenue in reducing NHS spend and demand on OA treatment and related issues.

## Data Availability

The original contributions presented in the study are included in the article/**Supplementary Material**, further inquiries can be directed to the corresponding author/s.
