# Supplementary material for: A scalable 12-week exercise and education programme reduces symptoms and improves function and wellbeing in people with hip and knee osteoarthritis
Source: Front Rehabil Sci. 2023 Apr 26;4:1147938. doi: 10.3389/fresc.2023.1147938 (PMC10169612; doi:10.3389/fresc.2023.1147938)
Supplement: Supplementary file 1 [file Table1.docx]

**Supplementary Table 1.** Demographic data

| **Combined** | | |
| --- | --- | --- |
|  | **Frequency** | **Percentage** |
| **Location of Pain** | | |
| Hips | 505 | 32 |
| Knees | 1055 | 66 |
| Hips & Knees | 33 | 2 |
| **Age** | | |
| 64 (9) | | |
| **Gender** | | |
| Male | 260 | 16 |
| Female | 1333 | 84 |
| **Employment Status** | | |
| Employed | 501 | 31 |
| Retired | 969 | 61 |
| Unable to work | 68 | 4 |
| Unemployed | 54 | 3 |
| NA | 1 | 0 |
| **Referral Pathway** | | |
| GP Practice | 23 | 1 |
| Other | 56 | 4 |
| Physio | 16 | 1 |
| Self | 1498 | 94 |
| **Hip** | | |
| **Age** | | |
| 64 (10) | | |
| **Gender** | | |
| Male | 72 | 14 |
| Female | 433 | 86 |
| **Employment Status** | | |
| Employed | 161 | 32 |
| Retired | 299 | 59 |
| Unable to work | 31 | 6 |
| Unemployed | 14 | 3 |
| **Referral Pathway** | | |
| GP Practice | 9 | 2 |
| Other | 20 | 4 |
| Physio | 7 | 1 |
| Self | 469 | 93 |
| **Knee** | | |
| **Age** | | |
| 62 (9) | | |
| **Gender** | | |
| Male | 183 | 17 |
| Female | 872 | 83 |
| **Employment Status** | | |
| Employed | 334 | 32 |
| Retired | 650 | 62 |
| Unable to work | 32 | 3 |
| Unemployed | 38 | 4 |
| **Referral Pathway** | | |
| GP Practice | 14 | 1 |
| Other | 36 | 3 |
| Physio | 9 | 1 |
| Self | 996 | 94 |
| **Hip and Knee** | | |
| **Age** |  |  |
| 63 (9) |  |  |
| **Gender** |  |  |
| Male | 5 | 15 |
| Female | 28 | 85 |
| **Employment Status** |  |  |
| Employed | 6 | 18 |
| Retired | 20 | 61 |
| Unable to work | 5 | 15 |
| Unemployed | 2 | 6 |
| **Referral Pathway** |  |  |
| Self | 33 | 100 |

Values for are presented as frequency and percentage, while age is presented as mean (SD). Combined: N=1593; Hip: N=505; Knee: N=1055; Hip & Knee: N=33.

**Supplementary Table 2.** Overview of education and exercise components of Nuffield Health Joint Pain Programme

| **Week** | **Educational Topic** | **Exercise Focus** | **Exercise Session 1** | **Exercise Session 2** |
| --- | --- | --- | --- | --- |
| 1 | Osteoarthritis overview | Cardiovascular | Circuit Training (examples include exercise bike, walking shuttle) | Low impact cardiovascular training class |
| 2 | Importance of Exercise | Joint Mobility | Circuit Training (examples include mobility stretches) | Dynamic flexibility class |
| 3 | Emotional Wellbeing | Joint Stability | Circuit Training (examples include wobble balance, single leg balance) | Core stability class |
| 4 | Perception of pain | Strength | Circuit Training (examples include squats, leg extension, shoulder press, chest press) | Strength training class: machine weights |
| 5 | Healthy eating | Cardiovascular | Circuit Training (examples include exercise bike, walking shuttle) | Step aerobics class |
| 6 | Goal checking | Joint Mobility | Circuit Training (examples include mobility stretches) | Aqua class |
| 7 | Exercise and programming | Joint Stability | Circuit Training (examples include wobble balance, single leg balance) | Yoga class |
| 8 | Pain management | Strength | Circuit Training (examples include squats, leg extension, shoulder press, chest press) | Strength training class: free weights |
| 9 | Weight management | Cardiovascular | Circuit Training (examples include exercise bike, walking shuttle) | Indoor cycling class |
| 10 | Sleep, rest, and recovery | Joint Mobility | Circuit Training (examples include mobility stretches) | Pool-based workout class |
| 11 | Planning and programming | Joint Stability | Circuit Training (examples include wobble balance, single leg balance) | Progressive stretching class |
| 12 | Goal setting and self-management | Strength | Circuit Training (examples include squats, leg extension, shoulder press, chest press) | Strength training class: compound movements |

**Supplementary Table 3.** General health scores at Weeks 0 and 12

| **Hip** | | | |
| --- | --- | --- | --- |
|  | Week 0 | Week 12 | Δ Week 0 to 12 |
| Systolic Blood Pressure | 140 (19) | 133 (16) | -7 (1.3)*** |
| Diastolic Blood Pressure | 81 (10) | 79 (10) | -3 (0.8)*** |
| Body Mass Index | 28.8 (4.5) | 28.5 (4.4) | -0.3 (0.1)*** |
| Fasting Glucose | 4.8 (1.6) | 4.8 (1.6) | 0 (0.2) |
| Resting Heart Rate | 70 (11) | 71 (11) | 1 (0.8) |
| Timed Up and Go | 11.0 (3.9) | 8.7 (2.9) | -2.2 (0.2)*** |
| Waist To Hip Ratio | 0.9 (0.1) | 0.9 (0.1) | 0 (0.01)** |
| **Knee** | | | |
|  | Week 0 | Week 12 | Δ Week 0 to 12 |
| Systolic Blood Pressure | 139 (18) | 134 (17) | -5 (0.9)*** |
| Diastolic Blood Pressure | 82 (11) | 80 (10) | -2 (0.6)*** |
| Body Mass Index | 29.1 (4.5) | 28.7 (4.4) | -0.3 (0.1)*** |
| Fasting Glucose | 4.8 (1.7) | 4.7 (1.6) | -0.1 (0.1) |
| Resting Heart Rate | 71 (12) | 72 (11) | 1 (0.6) |
| Timed Up and Go | 10.8 (4.1) | 8.3 (2.8) | -2.4 (0.2)*** |
| Waist To Hip Ratio | 0.9 (0.3) | 0.9 (0.1) | 0 (0.01)** |
| **Hip & Knee** | | | |
|  | Week 0 | Week 12 | Δ Week 0 to 12 |
| Systolic Blood Pressure | 138 (16) | 135 (15) | -3 (3.4) |
| Diastolic Blood Pressure | 86 (10) | 83 (8) | -3 (2.8) |
| Body Mass Index | 30.6 (8.1) | 30.6 (8.4) | 0 (1.0) |
| Fasting Glucose | 4.8 (2.2) | 4.8 (1.8) | 0 (0.5) |
| Resting Heart Rate | 74 (12) | 72 (14) | -2 (3.5) |
| Timed Up and Go | 13 (5.6) | 12.8 (5.7) | -0.2 (1.2)*** |
| Waist To Hip Ratio | 0.9 (0.1) | 0.9 (0.1) | 0 (0.01) |

Values for each timepoint are presented as mean (SD), while delta values are presented as mean (95% CI). Higher scores indicate a worse health state. Differences between time points were analysed using paired t-tests. ***p < 0.001, **p < 0.01, *p < 0.05. Systolic Blood Pressure: Hip: n=471, Knee: n=989, Hip & Knee: n =32; Diastolic Blood Pressure: Hip: n=473, Knee: n=989, Hip & Knee: n =32; Body Mass Index: Hip: n=342, Knee: n=630, Hip & Knee: n =5; Fasting Glucose: Hip: n=301, Knee: n=649, Hip & Knee: n =28; Resting Heart Rate: Hip: n=468, Knee: n=987, Hip & Knee: n =32; Timed Up and Go: Hip: n=464, Knee: n=984, Hip & Knee: n =30; Waist to Hip ratio: Hip: n=454, Knee: n=943, Hip & Knee: n =26; N values for Δ Week 0 to 12.

**Supplementary Table 4.** Self-report weekly physical activity level

| **Combined** | | | | | | |
| --- | --- | --- | --- | --- | --- | --- |
|  | Week 0 | % | Week 12 | % | Δ Week 0 to 12 | % |
| 1-2 Hours a week | 325 | 20 | 132 | 8 | 193 | -12 |
| 2-3 Hours a week | 307 | 19 | 338 | 21 | 31 | 3 |
| Less than 1 Hour a week | 304 | 19 | 18 | 1 | -286 | -18 |
| More than 3 Hours a week | 623 | 39 | 1045 | 66 | 422 | 27 |
| NA | 34 | 2 | 60 | 4 |  |  |
| **Hip** | | | | | | |
|  | Week 0 | % | Week 12 | % | Δ Week 0 to 12 | % |
| 1-2 Hours a week | 104 | 21 | 37 | 7 | -67 | -14 |
| 2-3 Hours a week | 104 | 21 | 100 | 20 | -4 | -1 |
| Less than 1 Hour a week | 97 | 19 | 7 | 1 | -90 | -18 |
| More than 3 Hours a week | 188 | 37 | 338 | 67 | 200 | 30 |
| NA | 12 | 2 | 23 | 5 | 11 | 3 |
| **Knee** | | | | | | |
|  | Week 0 | % | Week 12 | % | Δ Week 0 to 12 | % |
| 1-2 Hours a week | 209 | 20 | 90 | 9 | -119 | -11 |
| 2-3 Hours a week | 199 | 19 | 227 | 22 | 28 | 3 |
| Less than 1 Hour a week | 198 | 19 | 11 | 1 | -187 | -18 |
| More than 3 Hours a week | 428 | 41 | 691 | 65 | 263 | 24 |
| NA | 21 | 2 | 36 | 3 | 15 | 1 |
| **Hip & Knee** | | | | | | |
|  | Week 0 | % | Week 12 | % | Δ Week 0 to 12 | % |
| 1-2 Hours a week | 12 | 36 | 5 | 15 | -7 | -21 |
| 2-3 Hours a week | 4 | 12 | 11 | 33 | 7 | 21 |
| Less than 1 Hour a week | 9 | 27 | 0 | 0 | -9 | -27 |
| More than 3 Hours a week | 7 | 21 | 16 | 48 | 9 | 27 |
| NA | 1 | 3 | 1 | 3 | 0 | 0 |

Values for are presented as frequency and percentage. Combined: N=1593; Hip: N=505; Knee: N=1055; Hip & Knee: N=33.

**Supplementary Table 5.** Hip Osteoarthritis Outcome Score (HOOS) and Knee Osteoarthritis Outcome Score (KOOS) subscale scores at Week 0 and 12.

| **HOOS** | | | |
| --- | --- | --- | --- |
|  | Week 0 | Week 12 | Δ Week 0 to 12 |
| Pain | 55.1 (17.8) | 69.5 (19.0) | 14.4 (1.4) *** |
| Symptoms | 59.6 (18.4) | 70.7 (19.4) | 11.1 (1.5) *** |
| Function in Daily Living | 59.8 (19.4) | 74.4 (19.4) | 14.5 (1.4) *** |
| Function in Sport and Recreation | 42.0 (25.3) | 60.6 (24.4) | 18.6 (2.1) *** |
| Hip-related Quality of Life | 38.3 (19.8) | 54.6 (21.2) | 16.3 (1.6) *** |
| Global Score | 54.7 (17.3) | 69.3 (18.4) | 14.7 (1.3) *** |
| **KOOS** | | | |
|  | Week 0 | Week 12 | Δ Week 0 to 12 |
| Pain | 59.1 (17.5) | 71.9 (17.3) | 12.8 (0.9) *** |
| Symptoms | 61.6 (18.1) | 72.5 (17.7) | 10.9 (0.9) *** |
| Function in Daily Living | 62.6 (19.2) | 76.8 (17.6) | 14.2 (0.9) *** |
| Function in Sport and Recreation | 29.9 (24.6) | 48.6 (26.0) | 18.7 (1.4) *** |
| Knee-related Quality of Life | 36.9 (19.9) | 52.5 (19.5) | 15.6 (1.1) *** |
| Global Score | 55.3 (16.6) | 69.3 (16.4) | 14.0 (0.8) *** |
| **Hip & Knee: KOOS** | | | |
|  | Week 0 | Week 12 | Δ Week 0 to 12 |
| Pain | 54.1 (22.5) | 68.2 (23.3) | 14.2 (7.8) *** |
| Symptoms | 57.1 (18.4) | 72.8 (19.8) | 15.6 (7.2)*** |
| Function in Daily Living | 53.2 (23.5) | 72.5 (24.3) | 19.4 (8.4) *** |
| Function in Sport and Recreation | 31.2 (28.5) | 48.3 (28.4) | 17.2 (9.6)*** |
| Knee-related Quality of Life | 27.9 (20.8) | 53.6 (24) | 25.7 (7.7)*** |
| Global Score | 49 (20.9) | 66.9 (22.5) | 17.9 (7.2)*** |
| **Hip & Knee: HOOS** | | | |
|  | Week 0 | Week 12 | Δ Week 0 to 12 |
| Pain | 50.6 (21.0) | 63.0 (23.7) | 12.4 (7.8) ** |
| Symptoms | 45.8 (22) | 63.4 (23.1) | 17.6 (8.1)*** |
| Function in Daily Living | 48.6 (19.8) | 66.9 (23.9) | 18.3 (8.7) *** |
| Function in Sport and Recreation | 23.7 (16.8) | 54.7 (28.1) | 31 (14.1)*** |
| Hip-related Quality of Life | 32.8 (21.1) | 52.3 (25.7) | 19.5 (11.1)*** |
| Global Score | 44.6 (17.5) | 62.8 (23.1) | 18.2 (8.1)*** |

Values for each timepoint are presented as mean (SD), while delta values are presented as mean (95% CI). Higher scores indicate a better health state. Differences between time points were analysed using paired t-tests. ***p < 0.001, **p < 0.01, *p < 0.05. HOOS: N=505; KOOS: N=1055; Hip & Knee HOOS: N=26; Hip & Knee: KOOS: N=24.
